# Downregulation of blood serum microRNA 29 family in patients with Parkinson’s disease

**DOI:** 10.1038/s41598-017-03887-3

**Published:** 2017-07-14

**Authors:** Xiaochen Bai, Yilin Tang, Mei Yu, Lei Wu, Fengtao Liu, Jianliang Ni, Zishan Wang, Jinghui Wang, Jian Fei, Wei Wang, Fang Huang, Jian Wang

**Affiliations:** 1 The State Key Laboratory of Medical Neurobiology, the Institutes of Brain Science and the Collaborative Innovation Center for Brain Science, Shanghai Medical College, Fudan University, 138 Yixueyuan Road, Shanghai, 200032 China; 2 Department of Neurology, Huashan Hospital, Fudan University, 12 Wulumuqi Zhong Road, Shanghai, 200040 China; 3 Tongde Hospital of Zhejiang Province, 234 Gucui Road, Hangzhou, 310012 Zhejiang Province China; 4 School of Life Science and Technology, Tongji University, 1239 Siping Road, Shanghai, 200092 China; 5 Shanghai Research Center for Model Organisms, Pudong, Shanghai, 201203 China

## Abstract

There is currently no reliable and easily applicable diagnostic marker for Parkinson’s disease (PD). The aims of the present study were to compare the expression profiles of the microRNA29 family (miR-29s) in blood serum from patients with PD with healthy controls and to clarify whether the expression of miR-29s is correlated with disease severity, duration or L-dopa therapy and whether expression depends on the gender and age of patients. The levels of blood serum miR-29s in 80 patients with PD and 80 unaffected controls were assessed by reverse transcription-quantitative real-time PCR. The PCR products were confirmed by cloning and sequencing. Additionally, the expression of miR-7 in the blood serum from PD patients and control subjects was assessed. Serum miR-29 levels were significantly downregulated in PD patients compared to healthy controls. The serum miR-29 levels in female PD patients were markedly higher than in male PD patients. The expression of serum miR-29a and miR-29c expression tended to decrease with disease severity. Moreover, we found that serum miR-7 levels did not differ between PD patients and control subjects. Therefore, the reduction of serum miR-29 levels, particularly miR-29a and miR-29c, warrants further investigation of its potential serving as biomarkers for PD.

## Introduction

Parkinson’s disease (PD), which is the second most common neurodegenerative disease after Alzheimer’s disease (AD), affects up to 1% of people over the age of 60^1, 2^. Loss of dopaminergic neurons in the substantia nigra and the presence of proteinaceous inclusions termed Lewy bodies, which are primarily composed of fibrillar α-synuclein, are prominent features of PD^3^. Epidemiological studies have demonstrated a higher prevalence of PD in men than in women^4, 5^. The pathological mechanisms of PD are complex, and both genetic and epigenetic factors contribute to progressive neuronal death. MicroRNAs (miRNAs) are small non-coding RNAs of 20–25 nucleotides that mediate posttranscriptional gene repression of target RNA transcripts. The miRNA29 family (miR-29s) includes hsa-miR-29a and hsa-miR-29b-1, as well as hsa-miR-29b-2 and hsa-miR-29c, which are transcribed from two different gene clusters located on chromosome 7 and chromosome 1, respectively, of the human genome^6^.

Many studies have shown that miR-29s act as tumor suppressors in several types of cancer, although they can be oncogenic in other cancers^7, 8^. The involvement of miR-29s in the fibrosis of peripheral tissues has also been well documented^9^. In the central nervous system, miR-29s regulate neuronal maturation^10^ and dendritic spine morphology^11^. Dysregulation of miR-29s also has implications in aging^12^ and various neurological disorders such as AD^13^, Huntington’s disease^14^, amyotrophic lateral sclerosis^15^, multiple sclerosis^16^. The role of miR-29s in ischemia remains controversial^17–19^. In addition, two groups have reported evidence that miR-29s play a role in fine-tuning motor function^20, 21^. Collectively, miR-29s function in neuronal survival, proliferation, differentiation and plasticity.

However, the roles of miR-29s in PD remain unclear, and only a few studies have examined the expression of blood miR-29s in PD patients using microarray and quantitative real-time PCR (qRT-PCR)^22–25^. Additionally, in cellular and animal PD models, it has been found that dysregulation of some PD-related genes is attributable to the alteration in miRNAs. Among such particular miRNAs, miR-7, which is an evolutionarily conserved miRNA that represses the expression of α-synuclein, is associated with PD pathophysiology^26, 27^ and is downregulated in serum samples of PD patients^27^. In the present work, we measured the expression of serum miR-29s in a relatively large cohort of PD patients (n = 80) and healthy controls (n = 80). Additionally, all samples were used to detect the expression levels of miR-7.

## Results

### Blood serum miR-29s levels are significantly reduced in PD patients

**Figure 1:**
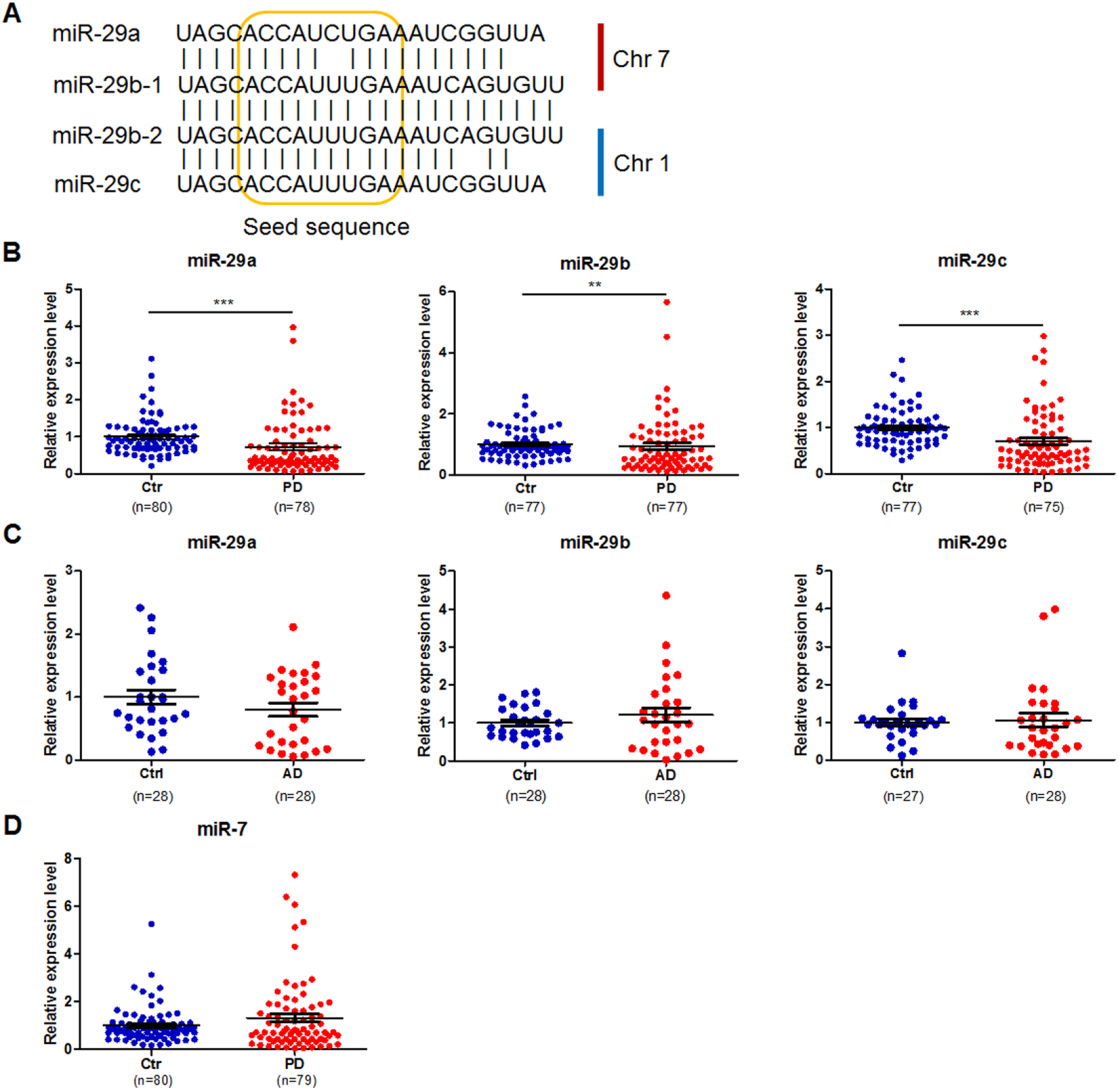
The alignment of human miR-29s (**A**) and the expression levels of miR-29s in the serum of control subjects and patients with PD (**B**) or AD (**C**) and the expression levels of miR-7 in the serum of controls and PD patients (**D**). Data are presented as the means ± SEM. Differences were analyzed by Mann-Whitney test. **p < 0.01 and ***p < 0.0001.

miR-29b-1 and miR-29b-2 (thereafter called miR-29b) have identical mature sequences, while miR-29a and miR-29c differ by one nucleotide (Fig. 1A). Mature miR-29s are highly conserved in humans, rats, and mice. Because circulating miRNAs in serum are sufficiently stable, they can serve as clinical biomarkers^28, 29^. Here, blood serum miR-29s levels were determined in 80 patients with PD and 80 controls.

The main demographic and clinical characteristics of the 80 idiopathic PD patients and 80 controls recruited in this study are summarized in Table 1. PD patients ranged from early to advanced PD (Hoehn & Yahr stage 1 to 3), and their ages were comparable across groups (average age-at-examination ± SD of 64.0 ± 5.8 years in patients and 63.3 ± 5.4 years in controls). qRT-PCR analysis was performed to examine the expression of blood serum miR-29s. The expression of miR-29a, miR-29b and miR-29c was not detected in samples from 2, 3 and 5 PD patients, respectively. Both miR-29b expression and miR-29c expression was not detectable in 3 control samples. The results were summarized in Fig. 1B, which revealed a marked reduction in serum miR-29s in PD patients compared to controls. To test the relative specificity of miR-29s in PD, blood serum miR-29 levels in 30 AD patients and 30 controls were measured. The primary demographic and clinical profiles of AD patients and control subjects are summarized in Table 2. Serum levels of miR-29s were comparable between patients with AD and their controls (Fig. 1C). Additionally, all samples from control subjects and PD patients were used to measure serum miR-7 levels, which showed that serum miR-7 expression was not altered (Fig. 1D).

**Table1:** Demographic and clinical profiles of PD patients and control groups.

	Controls	PD	Hoehn&Yahr stage I	Hoehn&Yahr stage II	Hoehn&Yahr stage III	p Value^a^
No. of subjects	80	80	29	25	26	—
Age, y	63.3 ± 5.4	64.0 ± 5.8	64.2 ± 5.9	63.0 ± 6.7	64.7 ± 5.0	0.777
F/M	32/48	32/48	12/17	12/13	8/18	0.807
Disease duration, mo	—	52.9 ± 52.2	23.7 ± 20.0	60.8 ± 63.7	77.8 ± 50.7^d^	0.001
UPDRS (motor)^b^	—	27.9 ± 14.0	16.6 ± 6.0	30.8 ± 9.2^d^	37.5 ± 15.6^e^	< 0.001
Levodopa equivalent dose (mg/day)	—	312.4 ± 319.2	196.7 ± 269.4	301.7 ± 300.9	451.8 ± 343.4^c^	0.030
No. of drug-naïve patients	—	19	10	8	1	—
MMSE	—	27.4 ± 2.5	28.2 ± 1.4	26.9 ± 2.3	27.0 ± 3.4	0.331

Abbreviations: PD = Parkinson disease; UPDRS = Unified Parkinson’s Disease Rating Scale; MMSE = Mini Mental State Examination. The data are presented as mean ± SD.

^a^Analysis of variance with the exception of chi-square for gender.

^b^Off-state motor ratings according to the UPDRS.

^c^p < 0.05 vs. Hoehn & Yahr stage I group.

^d^p < 0.01 vs. Hoehn & Yahr stage I group.

^e^p < 0.001 vs. Hoehn & Yahr stage I group.

**Table 2:** Demographic and clinical profiles of AD patients and control groups.

	AD	Controls	p Value
No. of subjects	30	30	—
Age, y	78.6 ± 9.5	42.6 ± 11.9	<0.001^a^
F/M	16/14	12/18	0.301^b^

Abbreviations: AD = Alzheimer Disease.

The data are presented as mean ± SD.

^a^p values were calculated using two-tailed Student’s t test.

^b^p values were calculated using chi-square test.

### Blood serum miR-29a and miR-29c tended to decrease with PD severity but not disease duration and UPDRS scores

**Figure 2:**
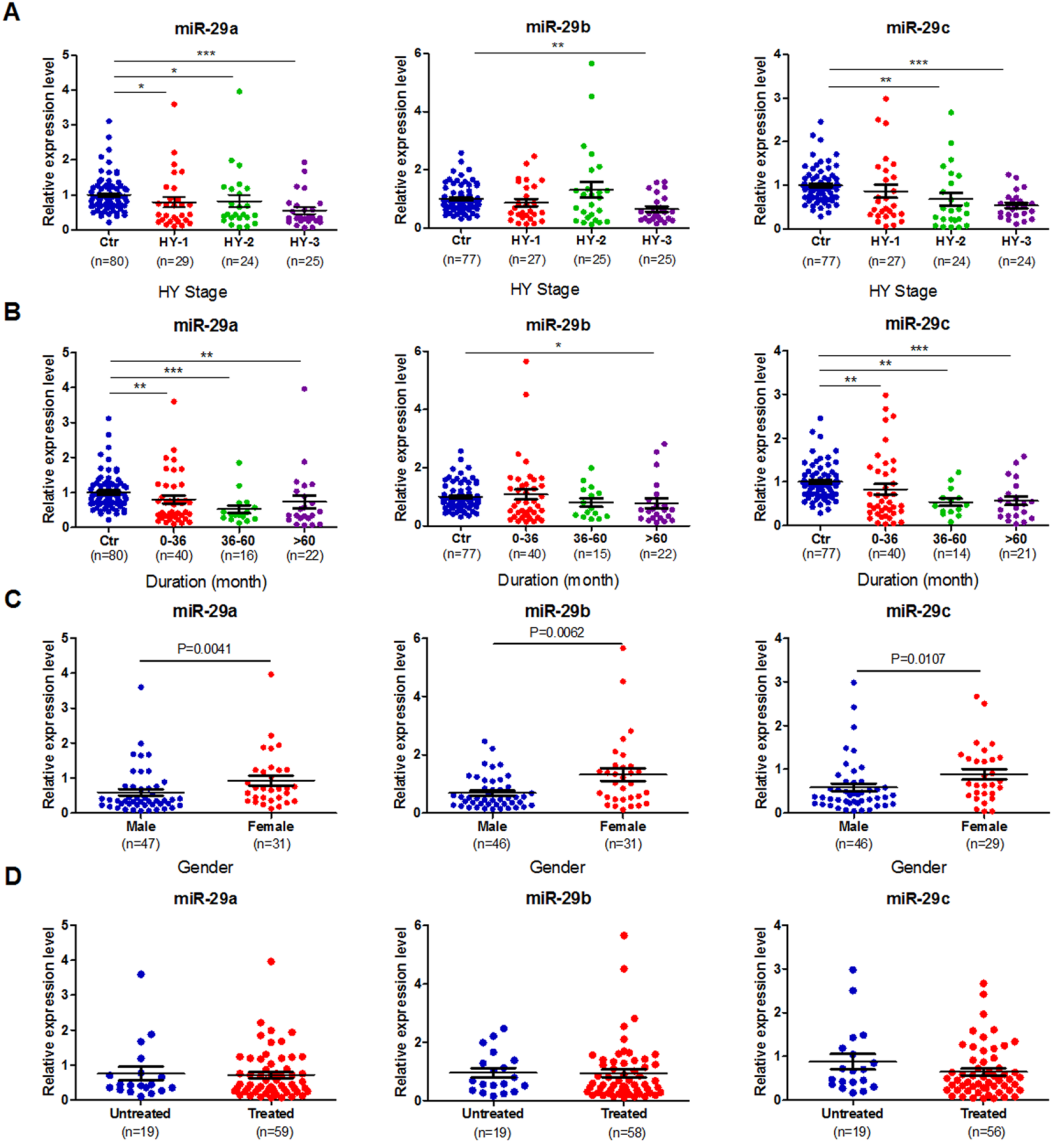
The expression levels of miR-29s in the serum of control subjects and patients with PD based on Hoehn & Yahr stages (**A**), disease duration (**B**), gender (**C**) and the expression levels of miR-29s in the serum of L-dopa-naïve PD patients and L-dopa-treated PD patients (**D**). Data are presented as the means ± SEM. Differences were analyzed by Kruskal-Wallis test in A and B or Mann-Whitney test in C and D. *p < 0.05, **p < 0.01 and ***p < 0.001.

The Hoehn & Yahr stages are widely used clinical standards for evaluating PD severity. Serum miR-29a and miR-29c tended to decrease with disease severity. The lowest expression of miR-29a and miR-29c was detected in HY-3 patients (Fig. 2A). Notably, there was a significant difference in serum miR-29b expression between control subjects and HY-3 patients. The lowest expression of miR-29b was detected in patients with disease durations longer than five years (Fig. 2B). However, there was no association between serum miR-29s and disease duration, and there was also no correlation between serum miR-29 expression and UPDRS score (Supplemental Table 1).

### Blood serum miR-29s show gender- but not age-dependent differences in PD patients

Although the serum miR-29 levels did not change with age in control subjects, the serum levels of miR-29a and miR-29c were markedly higher in females than in males (Supplemental Fig. 1). Similarly, in PD patients, miR-29 expression was significantly elevated in females (Fig. 2C). There were no age-dependent differences in the expression of miR-29s in patients with PD (Supplemental Table 1).

### Blood serum miR-29s in L-dopa-naïve PD patients are similar to those in L-dopa-treated PD patients

In this study, 80 patients with PD were divided into two categories: L-dopa-naïve patients (n = 19) and L-dopa-treated patients (n = 61). The effects of L-dopa therapy on the serum levels of miR-29s were evaluated. As shown in Fig. 2D, L-dopa therapy did not alter serum miR-29 expression (*P* = 0.9073 for miR-29a; *p* = 0.4286 for miR-29b; *p* = 0.1903 for miR-29c).

## Discussion

The profiles of blood miRNAs have been assessed in peripheral blood samples^24^, peripheral blood mononuclear cells^22, 23^, plasma^30^, and blood serum^25^ of idiopathic PD patients. Patient information is listed in Table 3 and includes L-dopa treatment, endogenous controls and the detected alterations in miR-29s. In this study, we focused on blood serum expression levels of miR-29s in 80 PD patients (including 19 L-dopa-naïve and 61 L-dopa-treated patients) and 80 matched controls. The serum levels of miR-29a and miR-29c were significantly decreased in PD patients and tended to reduce with disease severity. No alteration in serum miR-7 expression was detected in PD patients compared to control subjects. Our results are in agreement with those reported by Botta-Orfila *et al*.^25^ Serum miR-29b expression was also reduced in PD patients, although to a lesser extent. This result might have been due to the duplication of miR-29b in the human genome, which may affect its expression. Additionally, changes of miR-29s in PD serum are specific to some extents, as the miR-29 levels in AD serum do not differ from control serum.

**Table 3:** Summary of previous studies of miR-29s in patients with PD.

	Samples from subject Groups (n)	Age at inclusion (years; means ± SD)	Gender Men (%)	HY stage	Duration (years; means ± SD)	Normalizers	Results (relate to miR-29s)	Ref.
211(serum)	Controls(95)	67.22 ± 10.72	46.3	—	—	miR-17		25
	IPD(95)	67.7 ± 10.39	40	1–5	9.6 (CI4-12)	miR-106a	miR-29a/c ↓	
	LRRK2 PD(21)	61.83 ± 11.64	46.3	—	—		miR-29a/c ↓	
92(PBMCs)	Controls(36/10)	67 ± 10/67 ± 7	39/60	—	—			22
	L-dopa-treated(36)	68 ± 11	39	1–3	7 ± 6	RNU24	miR-29a ↑	
	Untreated (10)	68 ± 7	60	1–2.5	4 ± 3	Z30	miR-29a/b —	
23(blood)	Controls(8)	67 ± 8		—	—			24
	Untreated(8)	66 ± 6.7	50	1–2	3 ± 2.6	NA	miR-29a ↓	
	EOPD(7)	45 ± 8.7		1–3	7.2 ± 6.6		miR-29a —	
	Treated(4)^@^	—		—	—		miR-29a ↓	
32(PBMCs)	Controls(13)	64.38 ± 5.92	38.5	—	—	Microarrays		23
	PD(19)	65.11 ± 4.37	52.6	1–5	8.7 ± 5.1		miR-29b/c ↓	

Abbreviations: LRRK2 PD = Patients with LRRK2-associated Parkinson’s disease carrying the heterozygous G2019S mutation; EOPD = Early-onset Parkinson’s disease; HY stage = Hoehn & Yahr stage; PBMCs = Peripheral blood mononuclear cells.

NA = Not available.

^@^Selected previously untreated PD patients after 97 (±39) days of the levodopa/carbidopa treatment.

Gender, but not disease duration, UPDRS score, age or L-dopa treatment, affects serum miR-29 expression. It is well known that a higher incidence rate of PD is found among men, with the relative risk being 1.5 times greater in men than in women^31, 32^. The neuroprotective effects of estrogens, as well as gender-specific genetic factors, may account for this difference^32, 33^. Additionally, the significantly reduced serum levels of miR-29s in male PD patients are consistent with the increased risk of developing PD in men in our study.

In PD pathogenesis, mitochondrial dysfunction, oxidative stress, protein mishandling, and cell death together with epigenetic abnormality play central roles^1, 34^. Mature miR-29s have identical seed sequences at nucleotide positions 2–7, and the predicted target genes for miR-29s heavily overlap^9^. Candidate targets of miR-29s include: oxidative stress sensor *PARK7* (*DJ*-*1*), Parkin substrate *GPR37*, targets related to apoptotic processes *Puma*, *Bim*, *Bak*, *Bcl2*, *IGF1* and *AKT1*, microglial phagocytosis-related *CDC42*, and the epigenetic molecules *DNMT3A*, *DNMT3B* and *HDAC4*. miR-29a, and miR-29c were recently found to be downregulated in the same patients with idiopathic rapid eye movement behavior disorder after they were diagnosed with PD and dementia with Lewy bodies^35^. Therefore, the role of miR-29s in the pathogenesis of PD and the diagnostic potential of circulating miR-29s in PD patients warrant further study.

## Methods

### Subjects

Eighty patients with PD and thirty patients with AD were recruited from the Department of Neurology, Huashan Hospital, Fudan University, and Tongde Hospital, Zhejiang Province. PD subjects were clinically examined and diagnosed by two senior investigators of movement disorders according to the UK Brain Bank criteria^36^. Exclusion criteria included (1) clinical signs of possible atypical Parkinsonism; (2) secondary or iatrogenic Parkinsonism; (3) patients with cognitive impairment as assessed by the Mini Mental State Examination (MMSE); and (4) patients with hepatic and/or renal dysfunction. Patients were diagnosed with probable AD based on a comprehensive evaluation by two experienced subspecialty cognitive neurologists according to NINCDS-ADRDA^37^ criteria and their revision^38^. Exclusion criteria for AD patients included metabolic diseases, large vessel strokes, head injuries, severe psychiatric illness and neuro-developmental conditions. All participants provided written informed consent in accordance with the Declaration of Helsinki. The study was approved by the Human Studies Institutional Review Board, Huashan Hospital, Fudan University, and the Human Studies Institutional Review Board, Tongde Hospital, Zhejiang Province. All methods were performed in accordance with the relevant guidelines and regulations.

Of the 80 PD patients, 61 had received medication for PD; the remaining 19 had not been previously medicated. To standardize the data on medication use, we converted the dosages of PD medications into total daily levodopa-equivalent doses. Before clinical assessment, the subjects fasted overnight and did not take anti-Parkinsonian medications for at least 12 h. The severity and stage of the patient’s Parkinsonism was evaluated using the Unified Parkinson’s Disease Rating Scale (UPDRS) motor subscore^39^ and the modified Hoehn and Yahr stage^40^. Twenty-nine patients showed unilateral motor impairment only, classified as HY-1 stage; 25 patients had presented bilateral or midline involvement without impairment of balance, classified as HY-2 stage; and 26 patients exhibited with postural reflexes impairment, classified as HY-3 stage.

Overall, 110 age- and gender-matched volunteer control subjects were recruited. All control subjects had no history of neurologic/psychiatric disorders. The demographic and clinical data of patients and controls are summarized in Tables 1 and 2.

### Serum isolation and storage

The PD patients refrained from taking any anti-parkinsonian medications and fasted for at least 12 h before blood samples were taken. Control subjects fasted for 12 h before blood samples were taken. First, 5 ml of whole blood was collected between 8:00 and 9:00 a.m. in tubes without anticoagulant and was preserved for 30 minutes at room temperature according to the protocols from Parkinson Progression Marker Initiative (PPMI)^41^. Tubes were centrifuged at 1900× g for 10 minutes at 4 °C. Serum were removed, aliquoted (200 µl/tube), flash frozen, and stored at −80 °C.

### RNA extraction

Frozen sera were thawed at room temperature and centrifuged at 16000× g for 5 minutes at 4 °C. Then, 100 µl of supernatant was transferred to a new tube for the isolation of total RNA, including miRNAs, using miRNeasy Serum/Plasma Kit (Qiagen, Germany). A final 12 μl of the eluate was collected. To normalize for the miRNA content, each denatured sample was supplemented with 3.5 µl (1.6 × 10^8^ copies/μl working solution) synthetic *Caenorhabditis elegans* miR-39 (cel-miR-39), as described previously^42, 43^.

### Reverse transcription and quantitative real-time PCR

First, 5 µl of total RNA was reverse transcribed using a miRcute miRNA First-Strand cDNA Synthesis Kit (Tiangen, China). Subsequently, 2 µl of the product was used to detect miR29s expression by quantitative real-time PCR using a miRcute miRNA qPCR Detection kit (Tiangen, China). The PCR primer sequences were as follows: miR-29a (5′-TAGCACCATCTGAAATCGG-3′); miR-29b (5′-TAGCACCATTTGAAATCAGT-3′); miR-29c (5′-TAGCACCATTTGAAATCGG-3′) and miR-7 (5′-TGGAAGACTAGTGATTTTGTT-3′). Relative expression levels were calculated using the comparative ΔΔC_t_ method with cel-miR-39 as the normalizing control. Samples with Ct values above 35 were randomly picked to run on 2.5% agarose gels. After recovering and cloning, they were confirmed by sequencing.

### Statistical analysis

Data were presented as the means ± SEM. For group-wise comparisons, the Mann-Whitney test (2 groups) or Kruskal-Wallis test (n groups) was used as appropriate. The relationships between miR-29 expression and disease duration, UPDRS score and age were assessed in PD patients via a logistic regression analysis and analysis of covariance (ANCOVA) using SPSS 19.0 (Version 19.0; SPSS, Chicago, USA). The statistical analysis was performed using PRISM 5.0 (GraphPad Software Inc, USA). Significant differences were defined as *P* < 0.05.

## Electronic supplementary material

Supplemental Information
