# Supplementary material for: Downregulation of blood serum microRNA 29 family in patients with Parkinson’s disease
Source: Sci Rep. 2017 Jul 14;7:5411. doi: 10.1038/s41598-017-03887-3 (PMC5511199; doi:10.1038/s41598-017-03887-3)
Supplement: Supplementary file 1 — Supplemental Information [file 41598_2017_3887_MOESM1_ESM.pdf]

SUPPLEMENTAL INFORMATION

Downregulation of blood serum microRNA 29 family in patients with Parkinson's disease

Xiaochen Bai, Yilin Tang, Mei Yu, Lei Wu, Fengtao Liu, Jianliang Ni, Zishan Wang, Jinghui Wang, Jian Fei, Wei Wang, Fang Huang, Jian Wang

SUPPLEMENTAL TABLES

TABLE S1 Correlation analysis of between serum miR-29s expression and other predictors (disease duration, UPDRS score or age).

	Duration		UPDRS		Age	
	r	p	r	p	r	p
**miR-29a**	-0.123	0.283	-0.123	0.292	1.708(F)	0.054
**miR-29b**	-0.14	0.223	-0.022	0.852	-0.067	0.564
**miR-29c**	-0.17	0.145	0.471(F)	0.984	-0.220	0.058

SUPPLEMENTAL FIGURES

A

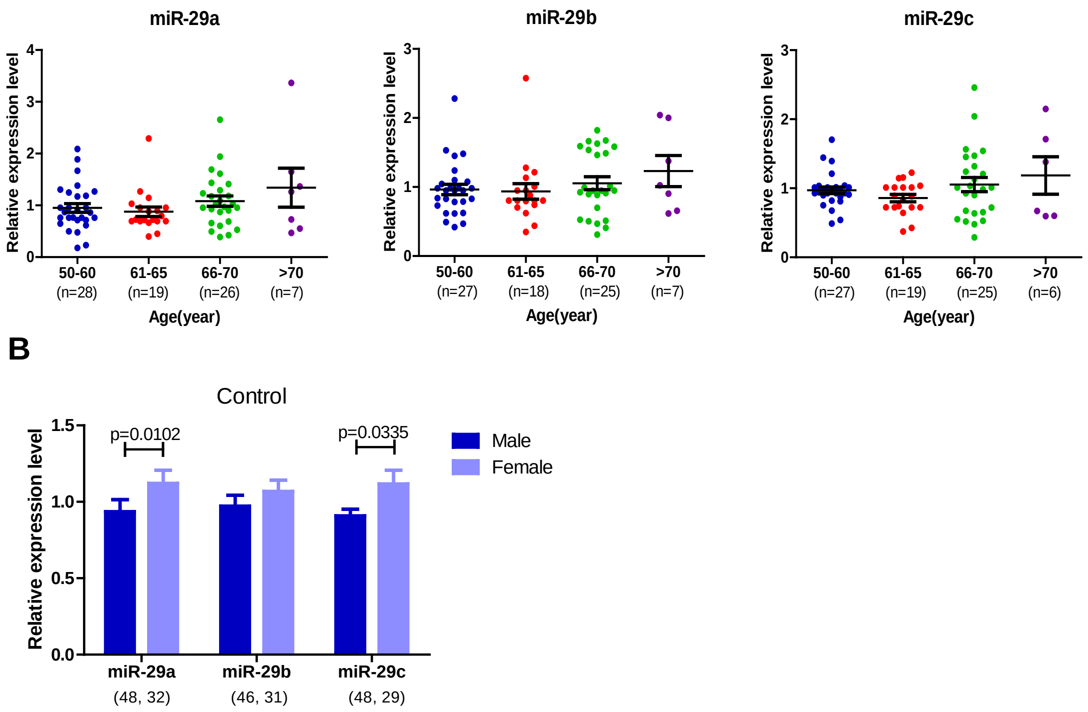

FIGURE S1. The serum miR-29s levels in the control subjects. (A) Serum miR-29s levels did not alter with the age; **(B)** Serum levels of miR-29a and miR-29c were markedly higher in females when compared to those in males.
